# Detection of Illicit Drugs by Trained Honeybees (*Apis mellifera*)

**DOI:** 10.1371/journal.pone.0128528

**Published:** 2015-06-17

**Authors:** Matthias Schott, Birgit Klein, Andreas Vilcinskas

**Affiliations:** 1 Institute of Phytopathology and Applied Zoology, Justus-Liebig-University of Giessen, Giessen, Germany; 2 Department Bioresources, Fraunhofer Institute for Molecular Biology and Applied Ecology (IME), Giessen, Germany; 3 Forensic Institute - Section Narcotics/Chemistry, State Office of Criminal Investigation Hessen, Wiesbaden, Germany; University of Cologne, GERMANY

## Abstract

Illegal drugs exacerbate global social challenges such as substance addiction, mental health issues and violent crime. Police and customs officials often rely on specially-trained sniffer dogs, which act as sensitive biological detectors to find concealed illegal drugs. However, the dog “alert” is no longer sufficient evidence to allow a search without a warrant or additional probable cause because cannabis has been legalized in two US states and is decriminalized in many others. Retraining dogs to recognize a narrower spectrum of drugs is difficult and training new dogs is time consuming, yet there are no analytical devices with the portability and sensitivity necessary to detect substance-specific chemical signatures. This means there is currently no substitute for sniffer dogs. Here we describe an insect screening procedure showing that the western honeybee (*Apis mellifera*) can sense volatiles associated with pure samples of heroin and cocaine. We developed a portable electroantennographic device for the on-site measurement of volatile perception by these insects, and found a positive correlation between honeybee antennal responses and the concentration of specific drugs in test samples. Furthermore, we tested the ability of honeybees to learn the scent of heroin and trained them to show a reliable behavioral response in the presence of a highly-diluted scent of pure heroin. Trained honeybees could therefore be used to complement or replace the role of sniffer dogs as part of an automated drug detection system. Insects are highly sensitive to volatile compounds and provide an untapped resource for the development of biosensors. Automated conditioning as presented in this study could be developed as a platform for the practical detection of illicit drugs using insect-based sensors.

## Introduction

Approximately 5% of the world’s adult population (~230 million people) used illicit drugs at least once in 2010, and 0.6% of the adult population (~27 million people) are considered to be ‘problem users’ [1]. Illicit drug use appears to be stable in the developed world, but is increasing in several developing countries. These drugs are directly responsible for 0.2 million deaths per year, with heroin and cocaine the major culprits [1]. The authorities attempt to combat drug abuse and drug-related crimes by detecting and confiscating illegal drugs in transit and prosecuting those involved in drug trafficking. This is usually achieved by using sniffer dogs to detect concealed drugs because such dogs have a detection threshold significantly lower than commercial analytical devices [2]. Sniffer dogs are sensitive and efficient biosensors, but their disadvantages include the cost and long duration of training, and the short duty cycles [3]. Additionally, because there is a social relationship between the dog and its handlers, reactions can be biased by the trainer or operator, which can lead to subjective false positive or negative responses [2].

Recreational cannabis use was recently legalized in two US states and is decriminalized in many others [4–6]. Therefore, a sniffer dog “alert” is no longer sufficient evidence to allow police searches without permission, a warrant, or additional probable cause [5]. The retraining of sniffer dogs to ignore cannabis is difficult and time consuming [3]. Trained insects have been proposed as alternative biosensors for illegal drugs [7, 8] because their antennae are the most sensitive natural organs discovered thus far for the detection of volatiles [9]. Insects can be produced and reared inexpensively, and they can be conditioned rapidly to react to specific volatiles [2, 3, 8]. The ability of insects to sense and learn odors varies from species to species. Therefore, protocols must be developed to screen different species for their suitability in drug-detection applications.

The perception of odors by insects begins when volatile odor molecules interact with odorant-binding proteins (OBPs) in sensory organs known as sensilla, which are located on the antennae. The odor is translated into an electrical signal in the dendritic membrane when the OBP-odor complex binds to an odor receptor, and the resulting action potential transduces the information to the antennal lobe. Here, the signals are filtered and translated via pattern recognition to prepare the incoming signals for reactions and learning [10–12]. Insect species suitable for specialized detection applications can therefore be identified by electroantennography (EAG), in which insect antennae are connected to two electrodes that amplify and record the signals induced when odorant receptors on the antennal dendrites interact with the corresponding OBP-odor complexes [13]. The antennal response in the presence of diluted samples can be used to establish a dose—response relationship, which confirms that genuine reception events have occurred in the antenna. Once it is confirmed that an insect species is physiologically able to sense an odor, conditioning studies can be used to determine whether the species can link the novel odor with a reliable and machine-readable behavioral reaction.

Experimental validation is required to confirm that insects perceive specific illicit drugs, but such experiments are difficult to conduct because access to reference samples is restricted by laws covering the possession of narcotics. The ability of three insect species, the European grapevine moth (*Lobesia botrana*), the Madagascar hissing cockroach (*Gromphadorhina portentosa*), and the western honeybee (*Apis mellifera*) to sense volatiles associated with illegal drugs was therefore investigated using a recently developed portable EAG device [13]. This allowed the measurements to be undertaken at the Police Laboratory for Criminal Technology in Hesse, Germany, and was used to determine the quantitative response of honeybee antennae when exposed to volatiles associated with pure and cut illegal drugs such as heroin, cocaine, amphetamine and cannabis. The same device was used to record antennal responses to dilution series of the same samples. In the final step, we used portable automated training chambers to assess the learning abilities of the honeybees. We tested the statistical power of our training method and estimated the number of bees needed for further training experiments and the design of biosensors.

## Materials and Methods

### Maintenance of insects

The European grapevine moth (*Lobesia botrana*) was reared and separated by sex as previously described [13]. Only males (2–5 days old) were used in this investigation, to avoid antennal responses elicited by the detection of natural pheromones released by females. Antennal preparation and integrity was assessed by monitoring the response to the pheromone main component (*E*,*Z*)-7,9-dodecadienyl acetate. The Madagascar hissing cockroach (*Gromphadorhina portentosa*) was reared in the laboratory at room temperature and was fed on dry flake fish food and apple pieces twice weekly. Second-instar larvae were used in this investigation. Foraging western honeybees (*Apis mellifera*) were collected at the entrance of a colony maintained at the Department Bioresources, Fraunhofer Institute for Molecular Biology and Applied Ecology, Giessen, Germany. The specimens used in the EAG experiment were kept in plastic boxes in groups of 10 and supplied with a 50% sucrose/water solution and pure water ad libitum. For the conditioning process, foraging workers from the same colony were caught using an artificial sugar solution feeder placed on the laboratory windowsill. After the conditioning and testing procedure, which takes 13 min to complete, the bees were marked and released. Marked bees were not used for subsequent experiments.

### Narcotics

The illicit narcotic substances were tested at the Kriminaltechnisches Institut Wiesbaden (the Forensic Science Institute of the German state of Hesse). Heroin, cocaine, amphetamine and cannabis were chosen because they are prominent representatives of the main drug categories listed in the United Nations World Drug Report 2014 [14]. We tested heroin at 0.2% and 47.3% cut with a mixture of caffeine and N-acetyl-p-aminophenol (paracetamol, acetaminophen) as found in street drugs, as well as a pure heroin-chloride standard. Cocaine was tested at purities of 20% (cut with lidocaine and a small amount of caffeine), 70% cut with levamisole as found in street drugs, and as a pure standard. The detection of amphetamines was investigated using a 16% sample of α-methylphenethylamine cut with caffeine and a 100% standard. The reaction to cannabinoids was tested with pure marijuana blooms. Caffeine was tested as a pure standard. Each sample is described in the manuscript using the percentage concentration of its narcotic ingredient.

### EAG screening

The tips of Pasteur pipettes were filled with a small amount of glass wool to prevent the ejection of the test substances and then rinsed with GC—MS grade hexane and acetone (both Sigma—Aldrich, St. Louis, USA) before heating to 200°C for 1 h. The pipettes were allowed to cool and ~100 mg of each test substance was placed inside. The insects were sedated with CO_2_ and one antenna was removed using a razor blade (*L*. *botrana*, *G*. *portentosa*) or microscissors (*A*. *mellifera*) as appropriate for the antennal morphology. The antennae were inserted into an antenna holder chip adapted to the morphology of each species [13]. After transferring the chip to the EAG apparatus [13] each antenna was tested with 1 μl of a substance or a solution known to elicit a strong response in the corresponding species (control compounds) to ensure the quality of the preparation. Each 1-μl drop was applied to a 0.5-cm^2^ filter paper (Schleicher & Schuell, Dassel, Germany) in a Pasteur pipette. Grapevine moth antennae were tested with the main pheromone component (*E*,*Z*)-7,9-dodecadienyl acetate (Trifolio-M, Lahnau, Germany) at a concentration of 1 ng/μl in acetone, cockroach antennae were tested with 1 ng/μl dodecylacetate (Sigma-Aldrich) in acetone, and honeybee antennae were tested with GC—MS grade hexane as a control compound. Nonfunctioning antennae were discarded. Antennal responses declined over time, as determined by repeated presentation of the appropriate control compounds. Once the response fell below 50% of the original amplitude, the antennae were discarded. Air puffs (1 s in duration generated by an electric valve) were fed through the pipette into a stream of cleaned and humidified air (flow rate 20 ml/min) that passed over the antennae. The EAG response was recorded via an amplifier and analog-to-digital converter (IDAC 2, Syntech, Kirchzarten, Germany) using the program Autospike v3.9 (Syntech). Each substance was measured using antennae from three different individuals of each species.

### Analysis of dose-dependent antennal responses

Dose-dependent antennal responses of honeybees were verified by dissolving ~10 mg of each substance in 1 ml pure acetone and preparing serial tenfold dilutions in pure acetone, yielding four log-dependent dilutions of 10^–0^ to 10^–3^. A 1-μl drop of each dilution was applied to a 0.5-cm^2^ filter paper in a Pasteur pipette as above, and the acetone was allowed to evaporate for 1 min at room temperature prior to each measurement. All sample series were measured from the lowest dilution to the highest, and each sample was tested on the antennae from at least five different honeybees. We used 1 μl of 1 ng/μl *cis*-3-hexen-1-ol (Sigma-Aldrich) diluted in acetone as a control to verify the preparations and antennal integrity. Antennae were discarded if the response fell below 50% of the initial response or if the baseline showed irregularities.

### Conditioning procedure

Honeybees were conditioned to avoid an odor and the success of conditioning was tested by automated measurement as previously described [15]. Individual workers were introduced into acrylic glass measurement chambers (148 x 20 x 6 mm) featuring a floor and ceiling with a metallic grid. The position and movement of the insects were tracked using 26 infrared LED and sensor pairs orthogonal to the walking path with a sampling rate of 16 Hz. The chambers were enclosed to avoid interference caused by daylight, room lighting and shadows cast by the experimenters. Two different odors were introduced from each end of the chamber by computer-controlled valves. The odor onset side was chosen by a computer program relative to the position of the bees so there was an opportunity to flee from the odor. The odor plume was removed from the middle of the chamber by suction. The air flow was adjusted as previously described [15]. The pure test substances were diluted 10^−3^ in mineral oil (Sigma-Aldrich) and 150 μl of the solution was pipetted onto a Sugi absorbent strip (Kettenbach GmbH & Co. KG, Eschenburg, Germany) and placed in a 2-ml plastic syringe (Henke-Sass, Wolf GmbH, Tuttlingen, Germany). A second syringe with a Sugi absorbent strip containing 150 μl blank mineral oil was also prepared. For each odor, a solenoid pinch valve switched the air flow from the syringe containing pure mineral oil to the syringe containing the test odor. The training protocol [15] included a 30-s inter-trial interval. Odor A was presented as a conditioned stimulus for 8 s, but 2 s after release a mild electric shock (10 V, 1.2 Hz, pulse-duration 200 ms) was applied as unconditioned stimulus (eliciting a reflex response) to the ground and ceiling of the half-chamber in which the odor was presented. Odor B was presented for 8 s with no unconditioned stimulus. The workers were confronted with punished odor A and unpunished odor B in the sequence ABBABAAB. Five minutes after the last conditioning, the bees were tested for their response to the odors without an unconditioned stimulus in the sequence ABBA. Three experiments were carried out (i) using pure heroin as odor A and *cis*-3-hexenol as odor B; (ii) the reciprocal, and (iii) mineral oil as both A and B (blank control). To evaluate the success of training, the attractance index was calculated from the walking path. The attractance index is an integral between the baseline and the function of the walking path of the bee between the odor onset and offset [15]. Data were analyzed using R v3.1.2 (The R Foundation for Statistical Computing, Vienna, Austria) and an analysis script as previously described [15].

### Data processing and statistical analysis

The EAG data were combined with the digital signal that indicates the opening of the valve and integrated using the software OriginPro v8.1 (OriginLab Corporation, Northampton, USA). Statistical analysis was carried out using R v3.1.2. EAG responses to the 10^–0^ samples were compared with the response to acetone using Student’s *t*-test with correction of the significance levels using the Holm-Bonferroni method [16]. The AI values were compared using a paired *t*-test in R v3.1.2.

## Results

### Initial EAG screening shows diverse antennal response profiles in different insect species

**Fig 1:**
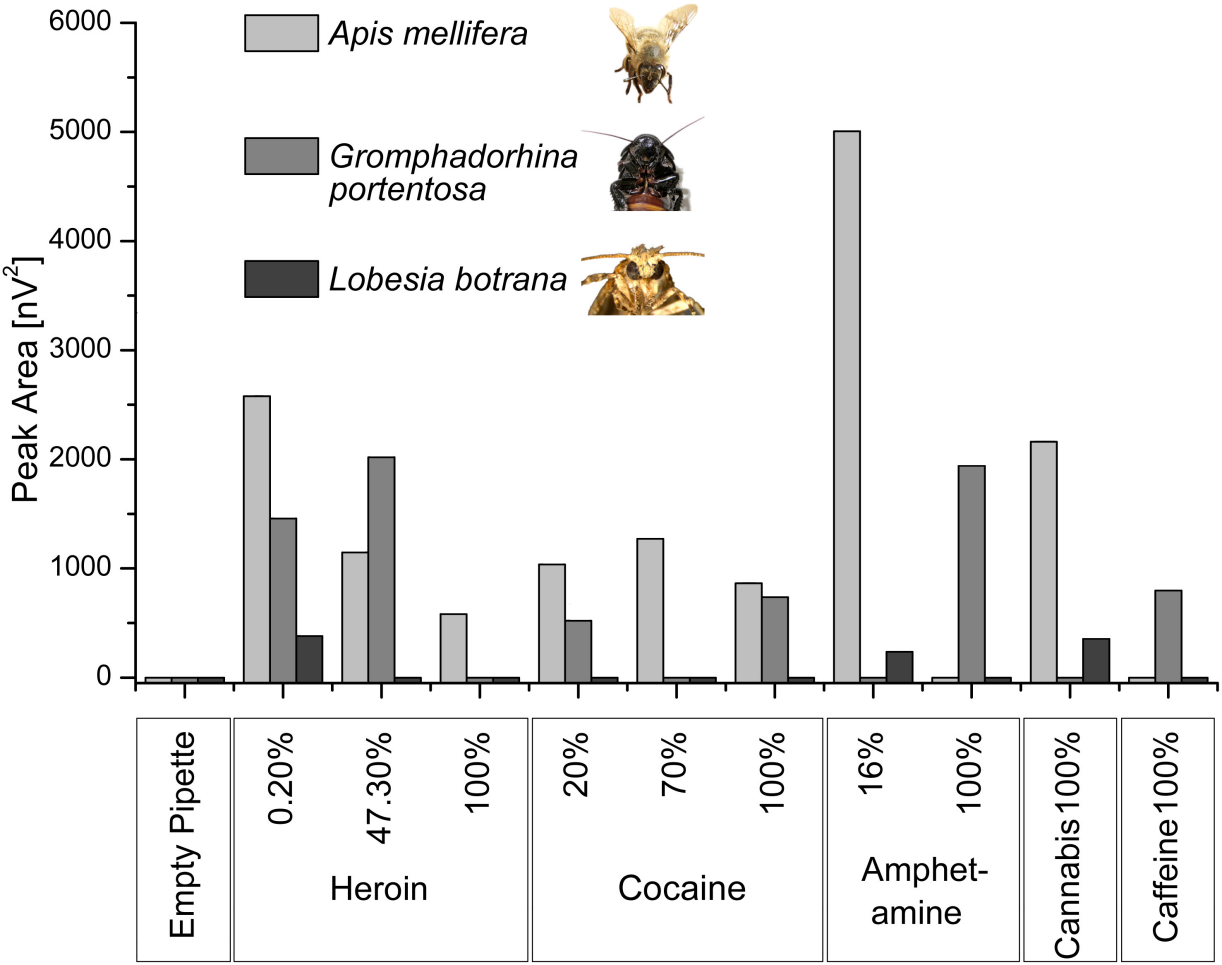
Absolute value of antennal responses from three insect species (western honeybee, hissing cockroach and grapevine moth) to drug volatiles (n = 3). The percentage value indicates the amount of pure drug relative to the whole sample (pure drug plus cutting agents).

An initial EAG screening experiment was carried out to evaluate the antennal responses of three insect species that are easy to rear (grapevine moth, hissing cockroach and western honeybee). Different response profiles were observed for each insect (Fig 1). We were particularly interested in the perception of pure illicit substances because reactions to contaminants such as cutting agents would generate ambiguous biosensor results. The impure heroin samples elicited measurable responses from the cockroach and honeybee antennae, but only the honeybee antennae responded to the pure heroin sample. Cocaine triggered EAG responses from the honeybee and cockroach antennae at all tested concentrations, but the response from the cockroach antennae was highly variable whereas the honeybee antenna showed evidence of a dose—response relationship. Amphetamine samples that were rich in cutting agents elicited a strong antennal response from the honeybee and a low response from the moth, but only the pure sample triggered a measureable response in the cockroach. The cannabis sample triggered responses in the honeybee and moth antennae, whereas the pure caffeine sample elicited a response solely from the cockroach antennae. The responses of the honeybee antennae towards pure heroin, cocaine and cannabis indicated an ability to perceive volatiles released by these samples, so it was necessary to establish whether the sensitivity of detection was adequate for biosensor applications.

### Honeybee antennae show a dose-dependent EAG response to cocaine and heroin

**Fig 2:**
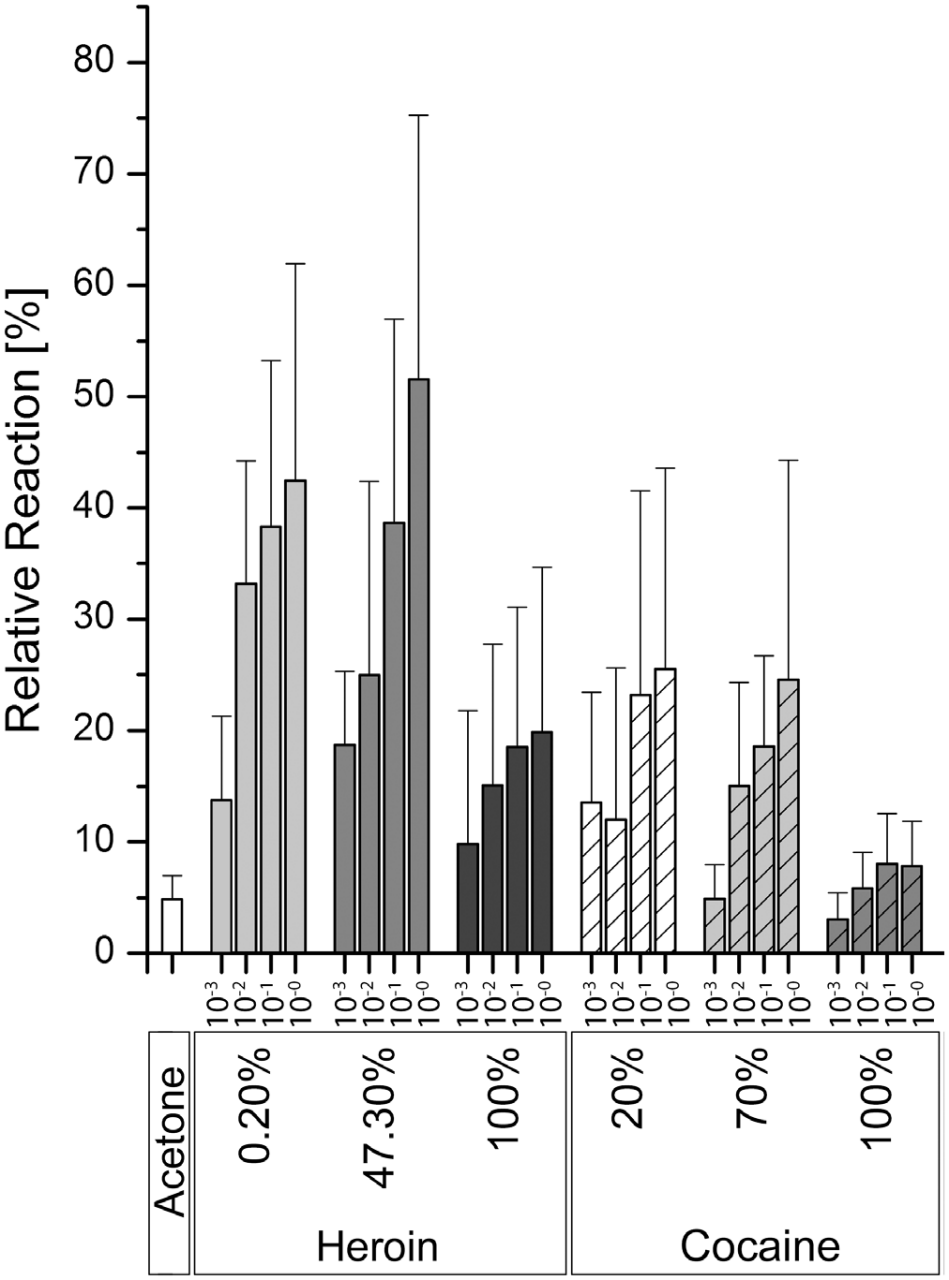
Honeybee antennal responses to four dilutions of the test samples in acetone, relative to the response to a *cis*-3-hexenol standard (concentration 1 ng/μl in acetone). Error bars indicate standard deviations (n = 10).

The initial findings described above were verified using a broader EAG dose—response experiment in which the drug samples were diluted in acetone and responses were recorded to four tenfold dilutions (10^0^ to 10^-3^). The strongest antennal responses were recorded in the presence of dilute solutions of heroin and cocaine (Fig 2). The dilutions that included cutting agents elicited stronger antennal responses than dilutions of the pure drugs. All pure and impure heroin and cocaine samples elicited sensillum reactions that differed significantly from the reaction to pure acetone, which was used as a control (S1 Table). The samples containing other drugs did not elicit responses that differed significantly from the control. Considering the dilution series, a clear dose—response relationship was observed for all heroin and cocaine samples, whether pure or cut (Fig 2). The amphetamine, cannabis and caffeine samples did not elicit dose-dependent reactions (S1 Fig).

### Honeybees can be conditioned to react predictably when exposed to pure heroin

**Fig 3:**
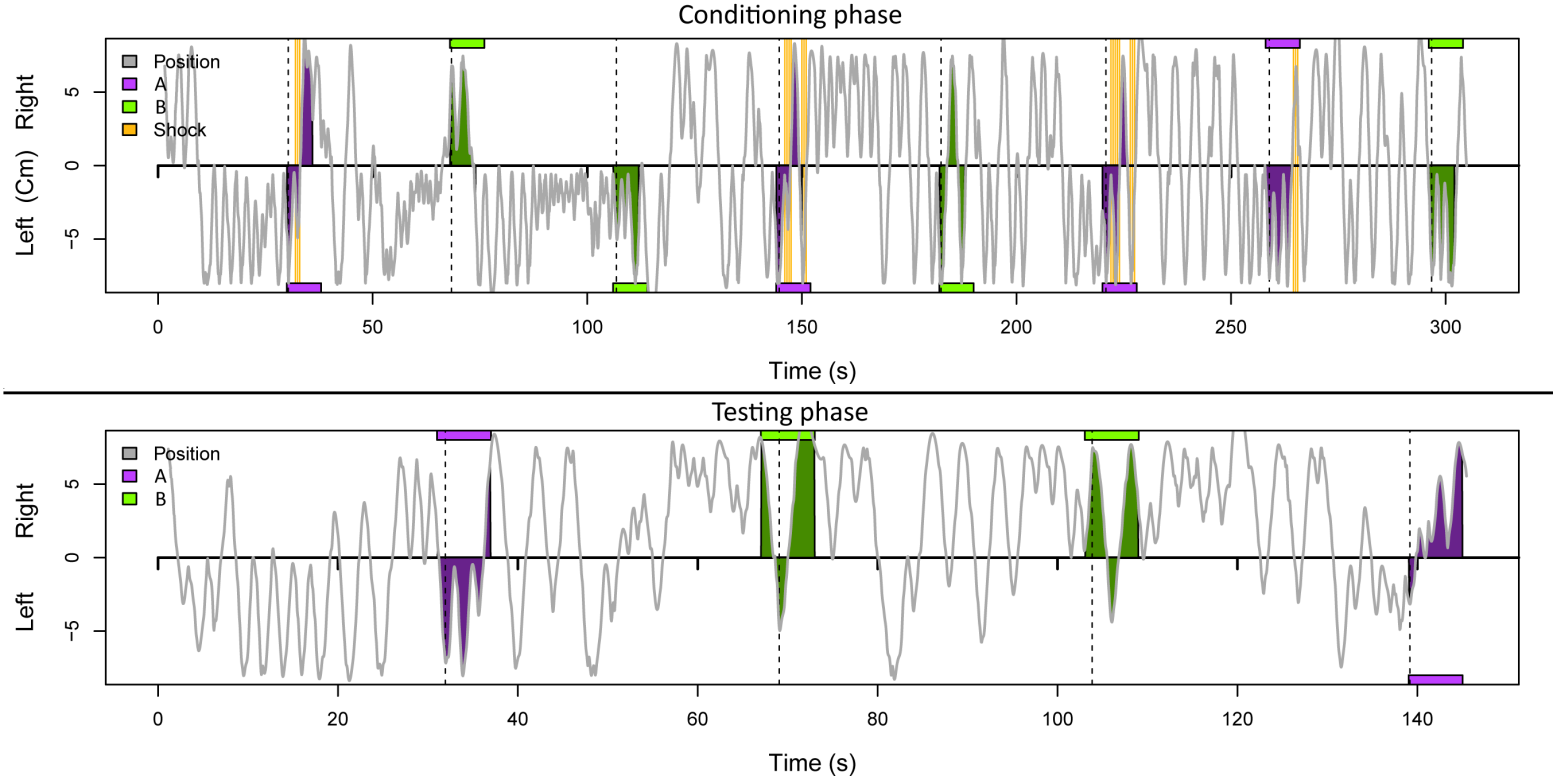
Example plot showing an individual honeybee conditioning experiment. The gray line indicates the walking trace from left to right in the conditioning chamber. Magenta and green boxes indicate heroin and *cis*-3-hexenol odor stimuli, respectively, and the side from which the odor was applied. Yellow lines indicate shock impulses. The upper plot shows the conditioning phase, the lower plot the test phase without shocks after a 5-min interval.

**Fig 4:**
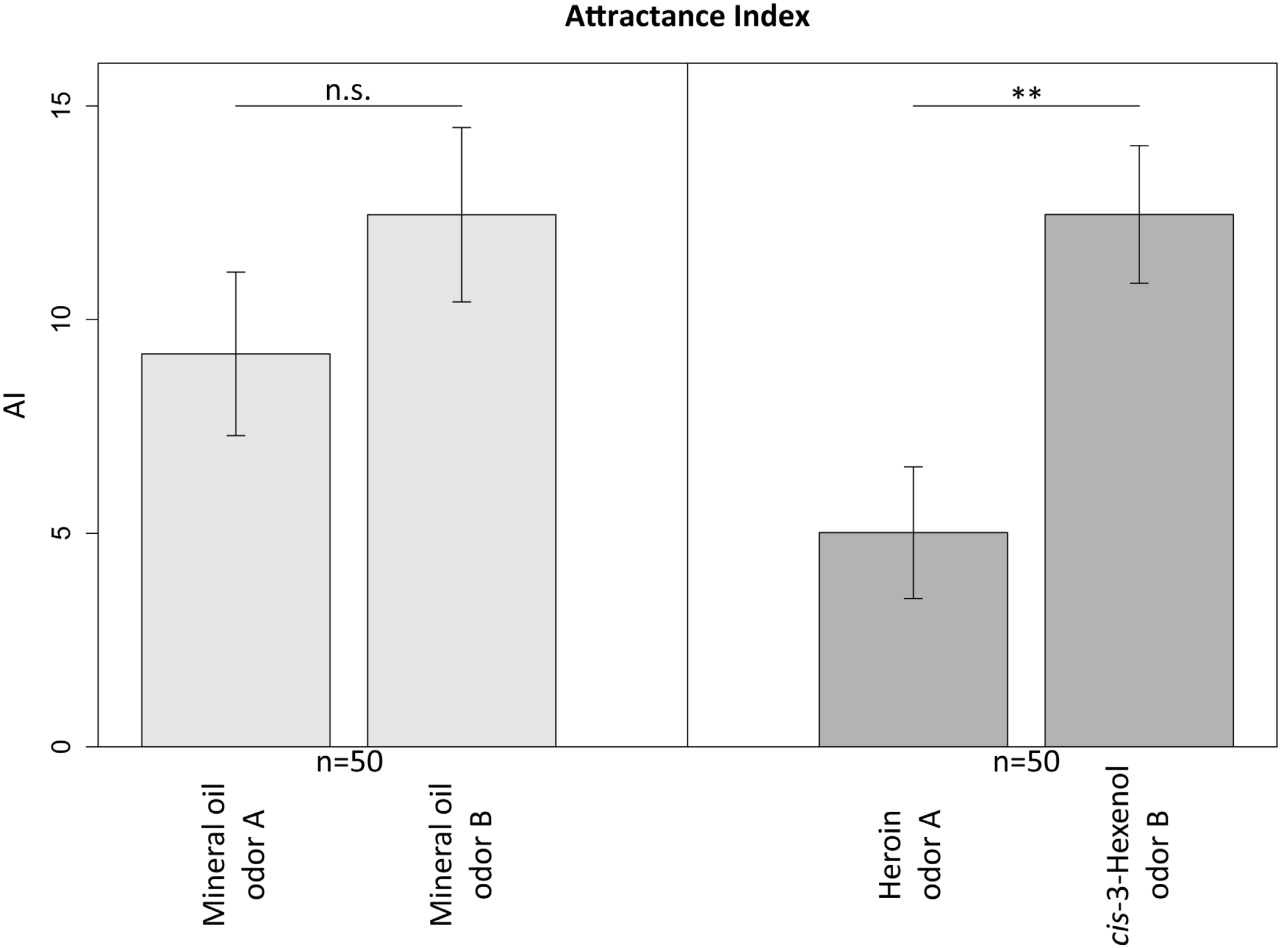
Mean attractance index values of the blank/blank and the heroin/*cis*-3-hexenol experiment in the test phase without an unconditioned stimulus. Odor A was the conditioned odor paired with the unconditioned stimulus in the conditioning phase, and odor B was presented always without the unconditioned stimulus. Error bars indicate standard errors (n = 50) and ** indicates a significant difference (p ≤ 0.001).

Honeybees were subjected to negative conditioning using an automated aversive conditioning arena. The success of training was evaluated by calculating the attractance index from the walking path as previously described [15] (Fig 3). A smaller attractance index value for the negatively-conditioned odor A (heroin) compared to control odor B (*cis*-3-hexenol, which was not combined with the unconditioned stimulus) is indicative of avoidance behavior. We observed a significant avoidance reaction (p ≤ 0.001) when honeybees were presented with the heroin scent after training with heroin as a conditioned stimulus (odor A) (Fig 4). No significant avoidance behavior was observed when honeybees were conditioned and presented with the mineral oil control odor A and B (p > 0.2). We then carried out a power analysis for a one-sided paired *t*-test with a true effect size of delta = 7.5, an upper limit of 340 for the variance, and a significance level of 5%, representing the probability of a type I error (false positive). This indicated that a population of 39 conditioned honeybees would be sufficient to obtain reliable results with a < 20% probability of a type II error (false negative) and this error rate could be reduced to < 5% by increasing the population size to 67. The reciprocal experiment, with *cis*-3-hexenol as the negatively-conditioned odor A and heroin as the unpunished odor B was conducted to determine whether the avoidance was learned or innate. This experiment indicated a significant avoidance reaction (p ≤ 0.01) when honeybees were presented with the *cis*-3-hexenol scent after conditioning (S2 Fig). After training and testing, all honeybees were marked and released. They behaved normally and returned frequently to the artificial feeder outside the laboratory.

## Discussion

Insect antennae are the most sensitive organs discovered thus far for the detection of volatile molecules [9] and insects have therefore been proposed as biosensors for many different types of odorants, in applications such as the detection of diseases, food contamination, explosive residues and drugs [2, 3, 7, 8, 12]. Insects and their isolated antennae are orders of magnitude more sensitive than the best artificial sensors and even sniffer dogs, and they are also smaller, less expensive, easier and quicker to train and more portable [2, 3, 7, 8, 12]. One drawback of insects is that their ability to sense particular chemicals is species-dependent, reflecting the availability of particular OBPs and the corresponding odorant receptors on the surface of olfactory neurons in the antennal sensilla. Therefore, the development of insect-based biosensors for illicit drugs depends on the identification of insect species that respond specifically and sensitively to the volatiles released from substances such as heroin and cocaine, regardless of the co-presentation of other odors that may arise from contaminants such as cutting agents. To evaluate the suitability of insect species as behavioral biosensors, an initial antennal response screening must be followed by a dose-dependent EAG study to confirm the initial findings, and finally conditioning experiments to prove that the odors can be associated with an unconditioned stimulus.

We tested three easily-reared insect species that are well known for their sensitive detection of volatiles—the grapevine moth, hissing cockroach and western honeybee. The response profiles of isolated antennae presented with a spectrum of illicit drug substances differed in a species-dependent manner, with the honeybee antennae showing the most promising results, i.e. specific responses to the presence of heroin and cocaine but not to the commonly used cutting agent caffeine (Fig 1). The absence of individual antennal responses does not necessarily rule out the perception of the corresponding volatiles but may indicate a low signal to noise ratio, which can be improved by serial measurements using multiple antennae. For example, up to four *Helicoverpa zea* antennae have been connected in series to achieve a 10-fold more sensitive response to *cis*-11-hexadecenal [17]. However, elaborate apparatus containing multiple antennae would be more difficult and time-consuming to set up on site, so we focused on a rugged and portable design which was more suitable for the limited time available at the police laboratories.

The initial experiment was suitable for the detection of diluted pure samples, but the application of such devices in the field would require the detection of much lower concentrations of each volatile. A more detailed dose—response test with diluted samples confirmed that honeybee antennae showed a dose-dependent reaction to heroin and cocaine (Fig 2). There was no significant reaction to amphetamine or cannabis (S1 Fig) even though both substances elicited a reaction in the initial screen (Fig 1). This demonstrates the need to carry out carefully-controlled dose—response studies to rule out aberrant responses, which in this case is reflected by the much lower and more realistic dilutions of the substances in the second experiment compared to the air puffs passing over the pure drug samples in the initial screen. The dose-dependent antennal reactions at low concentrations show that the honeybee is physiologically able to sense these substances (the minimal requirement for an effective insect-based drug sensor) whereas the more intense reactions towards the concentrations that include cutting agents may reflect the reactions of other antennal sensilla towards the contaminants. Synergistic and inhibitory effects of co-presented odors have been reported in honeybees [18]. Therefore, it is necessary to study the impact of different cutting agents on the sensitivity of honeybee antenna in further experiments. Nevertheless, the current iteration of the detector was able to achieve a dose-dependent response when less than 10 ng of the active compound was applied to the filter paper. The vapor pressures of cocaine and heroin are extremely low [19] and the volatile components are diluted even further by the air stream, so our data suggest that even minute quantities of the drug are sufficient to trigger the honeybee antennal response. Further studies are required to identify the volatile component that triggers the response, i.e. whether the drug itself or a decomposition product interacts with the odorant receptors.

Having established that honeybee antennae can specifically detect low quantities of heroin and cocaine, we carried out an aversive conditioning process to train living honeybees to avoid a heroin scent. The conditioned insects (heroin odor presented with unconditioned stimulus) displayed significant avoidance behavior in the presence of heroin, but not when exposed to the control substance *cis*-3-hexenol. In the reciprocal experiment, the conditioned insects displayed significant avoidance behavior in the presence of *cis*-3-hexenol but not heroin, confirming that the avoidance was conditioned rather than innate.

Power analysis indicated that a sensor device containing 40 free-walking, monitored, conditioned honeybees would be sufficient to yield reproducible behavioral responses in the presence of heroin with false positive rates of less than 5% and false negative rates of less than 20%. Therefore, a suitable device would contain 40 honeybees in behavioral monitoring chambers into which the test scent could be drawn, and avoidance behavior would trigger an “alarm” equivalent to the analogous signal given by sniffer dogs. The aversive conditioning method uses free-walking insects that are automatically trained rather than the labor-intensive proboscis extension reflex (PER) method with individually restrained and manually fed bees. In the PER method, the response is highly dependent on the condition of the honey bees, and insects that do not respond to the unconditioned stimulus or that die while being restrained must be excluded [20]. Fewer insects are needed in the aversive training chamber because the frequency of non-responding and dead insects is much lower [15]. The practical application of honeybee-based detectors also depends on consistent year-round performance. The comparative testing of PER-conditioned winter and summer worker honeybees has shown that even in long-term memory experiments, winter bees still recognized a conditioned odor but the summer bees performed more efficiently [21].

Further optimization of the conditioning parameters as well as a more symmetric training protocol could achieve reproducible results with even smaller populations of honeybees. For example, the odor and stimulus onset is not only timed, but the position of the honeybees in the chamber determines whether they have an opportunity to avoid the odor. Individual honeybees that happen to be in the middle of the chamber when the odor is released cannot display genuine avoidance behavior and learning cannot occur when they move directly away from the odor, which is then only present in the opposite site of the chamber. This is a rare occurrence but can be observed in Fig 3 during the final presentation of odor A in the training phase.

Although the other species we tested were not suitable for the detection of heroin and cocaine, the initial screening results suggest that they may nevertheless form the basis of useful sensors (Fig 1). Male grapevine moths were chosen because they are easy to rear and the excised antennae function for up to 10 h for EAG measurements [13]. Although the antennal response to drug volatiles was weak compared to the pheromone trace used as a standard, the initial screen suggested that grapevine moths may be useful for the detection of cannabis. Similarly, the cockroach antennae only reacted to amphetamine and caffeine. The amphetamine response occurred at 100% but not 16% suggesting we had either reached the detection threshold or that the cutting agents caused some form of receptor inhibition, as previously suggested [22]. If the cockroach antennae detect low amounts of amphetamine, whole insects could be used as a conditioned biosensor, similar to the method used to interpret the learning skills of restrained/immobilized *Periplaneta americana* cockroaches by monitoring their antennal movements [23].

Although the honeybee shows potential as a conditioned biosensor for the detection of specific drugs, the power of an insect-based drug detector platform could be increased by using several different species with diverse response profiles. For example, a multi-chamber device containing honeybees and cockroaches could be used in airports to screen luggage for heroin, cocaine and amphetamines, with air from the luggage drawn over the insects and “alarm” signals used to select luggage for more detailed investigation. Such a device could be used to support the activities of sniffer dogs by providing a more specific readout for particular classes of drugs.

## Supporting Information

S1 Fig Honeybee antennal reactions to four dilutions of the test samples in acetone, compared to a *cis*-3-hexenol standard (concentration 1 ng/μl in acetone). Error bars indicate standard deviation (n = 10).
(TIF)

S2 Fig Mean attractance index values of the *cis*-3-hexenol/heroin experiment in the test phase without an unconditioned stimulus. Odor A was the conditioned odor that was paired with the unconditioned stimulus in the conditioning phase, whereas odor B was presented always without the unconditioned stimulus. Error bars indicate standard error (n = 30) and * indicates a significant difference (p ≤ 0.01).
(TIF)

S1 Table Pairwise Student’s t-test comparisons of antennal reactions to illicit drugs (100 samples) compared with acetone controls, showing p values corrected using the Holm—Bonferroni method (p < 0.05). Bold numbers indicate statistically significant differences.
(DOCX)
