# Supplementary figures and images for: Detection of Illicit Drugs by Trained Honeybees (Apis mellifera)
Source: PLoS One. 2015 Jun 17;10(6):e0128528. doi: 10.1371/journal.pone.0128528 (PMC4471073; doi:10.1371/journal.pone.0128528)

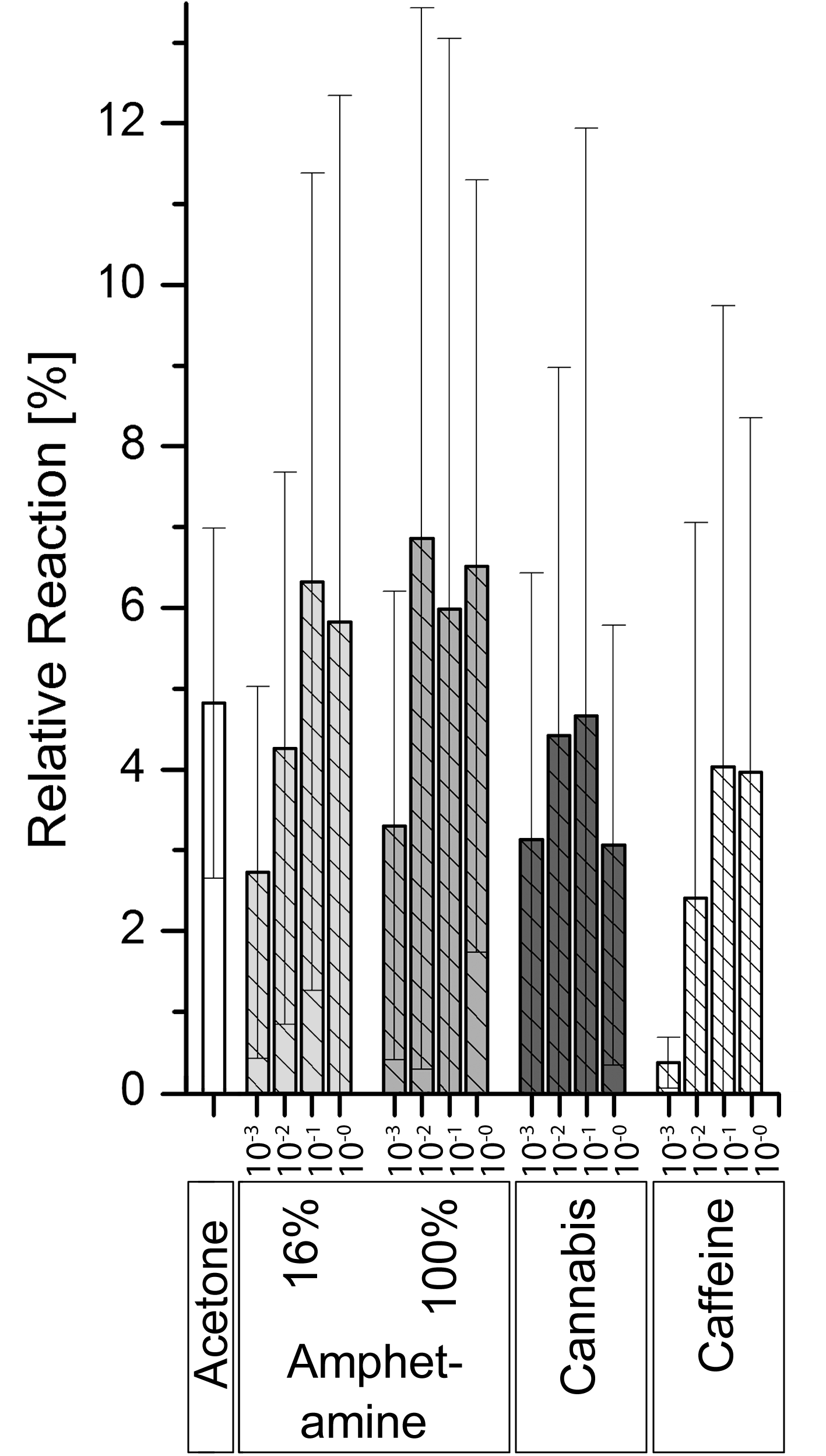

Supplement: S1 Fig — Error bars indicate standard deviation (n = 10). (TIF) [file pone.0128528.s001.tif]

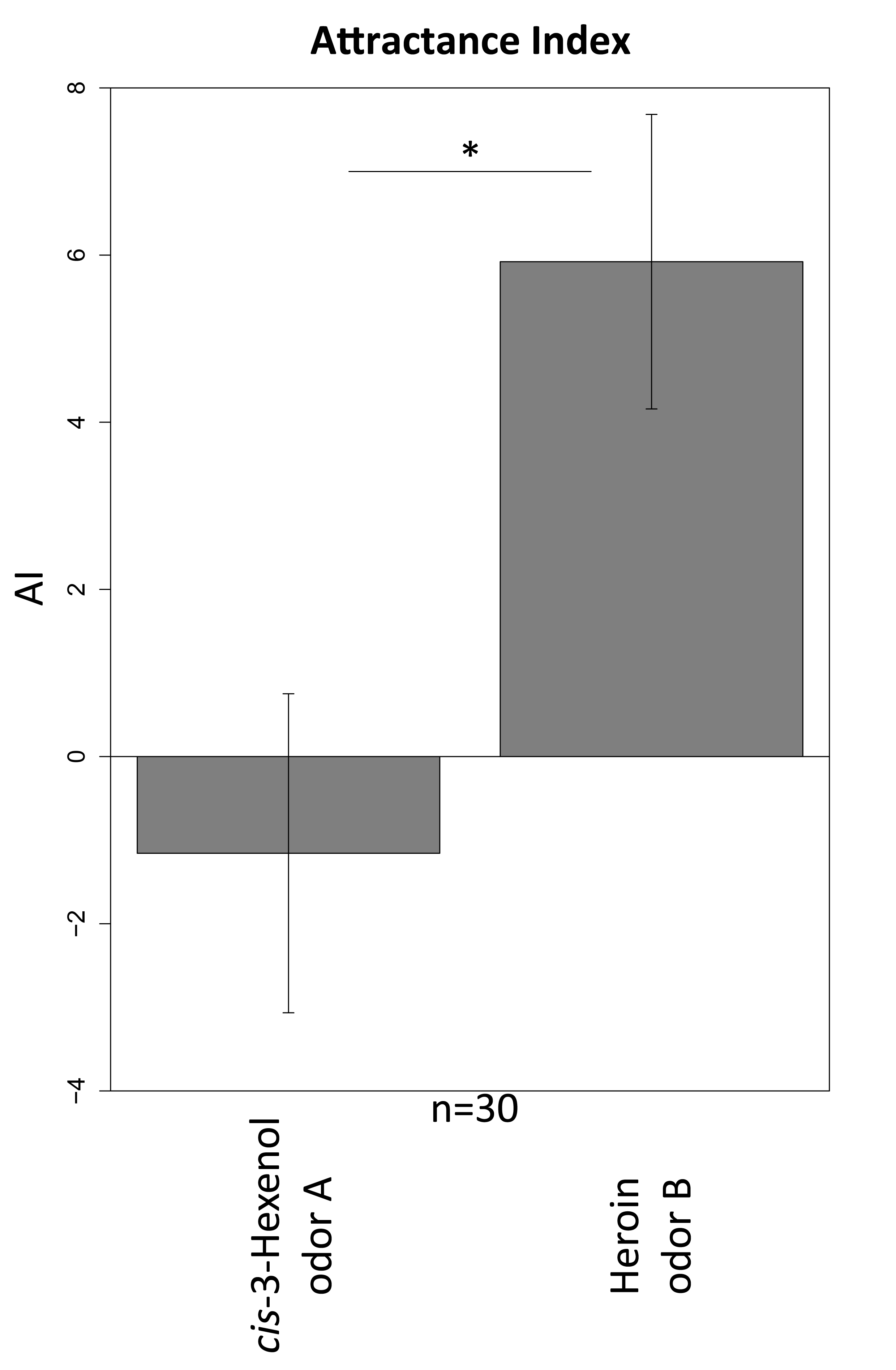

Supplement: S2 Fig — Odor A was the conditioned odor that was paired with the unconditioned stimulus in the conditioning phase, whereas odor B was presented always without the unconditioned stimulus. Error bars indicate standard error (n = 30) and * indicates a significant difference (p ≤ 0.01). (TIF) [file pone.0128528.s002.tif]
