# Supplementary material for: Detection of Illicit Drugs by Trained Honeybees (Apis mellifera)
Source: PLoS One. 2015 Jun 17;10(6):e0128528. doi: 10.1371/journal.pone.0128528 (PMC4471073; doi:10.1371/journal.pone.0128528)
Supplement: S1 Table — Bold numbers indicate statistically significant differences. (DOCX) [file pone.0128528.s003.docx]

| **Substance** | **t value** | **p value** |
| --- | --- | --- |
| Heroin 0.2% | -4.9407 | **0.015626** |
| Heroin 47.3% | -6.1496 | **0.017735** |
| Heroin 100% | -5.4186 | **0.016866** |
| Cocaine 20% | -5.4926 | **0.021416** |
| Cocaine 70% | -5.1424 | **0.01356** |
| Cocaine 100% | -2.9708 | **0.03113** |
| Amphetamine 16% | -1.9311 | 0.1257 |
| Amphetamine 100% | -2.3671 | 0.07706 |
| Cannabis | -0.4066 | 0.7051 |
| Caffeine | -0.4989 | 0.6441 |
